# Knowledge, attitudes, and practices of gouty arthritis in the general population aged > 30

**DOI:** 10.1186/s12909-024-05690-x

**Published:** 2024-07-19

**Authors:** Min Zhao, Jie Jian, Dong Yang, Honggang Sun, Ling Liu, Zhiyuan Yan, Yun Ma, Yugang Zhao

**Affiliations:** Chengdu Bayi Orthopedic Hospital, China RongTong Medical Healthcare Group Co. Ltd, No.3 Wudu Road, Qingyang District, Chengdu, Sichuan China

**Keywords:** Adults, Cross-sectional study, Gouty arthritis, Knowledge, Attitudes, Practices

## Abstract

**Background:**

A knowledge of gouty arthritis could help in the primary prevention of the disease development and lead to an early diagnosis if it occurs. This study investigated the knowledge, attitudes, and practices (KAP) toward gouty arthritis in the general population > 30 years old.

**Methods:**

This web-based cross-sectional study was conducted among the general population > 30 years old between January and March 2023 in Chengdu, Sichuan. The questionnaire was designed by the investigators based on the available guidelines (Cronbach’s α = 0.846). A score above 70% indicated good knowledge, a positive attitude, and proactive practice. Multivariable and structural equation modeling (SEM) analyses were performed to analyze the factors influencing KAP.

**Results:**

A total of 537 questionnaires were included. The knowledge, attitudes, and practices scores were 13.12 ± 6.41, 25.28 ± 3.97, and 45.25 ± 5.77, respectively. Female (OR = 0.47, 95%CI: 0.31–0.71, *P* < 0.001), suburban living (OR = 0.18, 95%CI: 0.04–0.78, *P* = 0.022), heads of institution/organization and professional and technical staff (OR = 2.04, 95%CI: 1.23–3.39, *P* = 0.006), and an income of < 2,000 yuan (OR = 0.35, 95%CI: 0.14–0.85, *P* = 0.021) were independently associated with knowledge. Female (OR = 2.17, 95%CI: 1.43–3.30, *P* < 0.001), age (OR = 1.03, 95%CI: 1.01–1.05, *P* = 0.001), college and above education (OR = 2.26, 95%CI: 1.16–4.41, *P* = 0.017), an income of 5,000–10,000 yuan (OR = 2.05, 95%CI: 1.27–3.31, *P* = 0.003), and an income of > 10,000 yuan (OR = 2.07, 95%CI: 1.12–3.81, *P* = 0.020) were independently associated with attitudes. Attitude (OR = 1.31, 95%CI: 1.23–1.40, *P* < 0.001), female (OR = 1.62, 95%CI: 1.01–2.58, *P* = 0.044), and age (OR = 1.02, 95%CI: 1.00-1.04, *P* = 0.016) were independently associated with practices. The structural equation modeling analysis showed that knowledge directly influenced attitude (β=-0.10, *P* < 0.001) and indirectly influenced practice (β=-0.07, *P* < 0.001), and attitude directly influenced practice (β = 0.68, *P* < 0.001).

**Conclusion:**

The general population over 30 years old had inadequate knowledge, unfavorable attitudes, and less proactive practices toward gouty arthritis. Targeted interventions should focus on enhancing knowledge about gout and promoting positive attitudes toward its management.

**Supplementary Information:**

The online version contains supplementary material available at 10.1186/s12909-024-05690-x.

## Introduction

Gouty arthritis is a chronic disease characterized by recurrent attacks of severe joint pain and swelling (gout flare) due to an inflammatory reaction directed against monosodium urate (MSU) crystals [1–4]. When gout is improperly managed, chronic joint inflammation may develop, as well as tophi, which can damage bone and cartilage permanently [1–4]. The deposition of MSU in the joint cavity activates inflammatory cytokines, inducing the accumulation of macrophages and neutrophils, which leads to gouty arthritis [5, 6]. Patients with gouty arthritis have high rates of comorbidities, which may complexify management [3]. The reported worldwide prevalence of gout ranges from 0.1 to 10%, and gouty arthritis is the most common inflammatory arthritis, particularly in males [3, 7]. Although most patients with asymptomatic hyperuricemia do not develop symptomatic gouty arthritis, MSU crystals can deposit on the cartilage surface [3, 4]. Multiple factors can increase the risk of hyperuricemia and gouty arthritis, including fructose or high-purine foods, alcohol consumption, acute or chronic kidney disease, and medications (e.g., low-dose aspirin, β-blockers, diuretics (except potassium-sparing diuretics), and anti-tuberculosis drugs like pyrazinamide and ethambutol) [2, 3]. A gout flare is an acute joint inflammation response to MSU crystals [3]. After a flare, the transition from asymptomatic hyperuricemia to gout has occurred, generally considered a lifelong diagnosis [2–4]. Gouty arthritis is a chronic condition [8]. The secondary prevention of acute gouty attacks and the exacerbation of gouty arthritis includes modifying risk factors (avoiding or decreasing high-purine foods, fructose, alcohol, and diuretics and managing obesity) and the long-term use of urate-lowering medications [1, 3, 9]. Urate lowering may benefit some gout comorbidities, although this is still being studied [3, 4].

Given that gouty arthritis is a public health issue in some populations with a high incidence and prevalence [3, 7] and since gouty arthritis is associated with modifiable risk factors (e.g., alcohol consumption and fructose and high purine foods), a knowledge of gouty arthritis could help in the primary prevention of gouty arthritis development and lead to an early diagnosis if it occurs. The knowledge, attitudes, and practices (KAP) methodology is a structural survey method that allows the identification of gaps that constitute barriers to the appropriate implementation or performance of a specific subject in a specific population [10, 11]. Previous studies identified knowledge gaps in primary practitioners [12–14]. A study from Pakistan revealed that many patients were suffering from gout in ignorance of what gout was and of the proper treatments [15]. No KAP data on gout or gouty arthritis are available in China. Therefore, this study aimed to examine the KAP of gouty arthritis in the general Chinese population > 30 years old.

## Methods

### Study design and participants

This web-based cross-sectional study was conducted among the general population > 30 years old between January and March 2023 in Chengdu, Sichuan. The inclusion criteria were 1) > 30 years old and 2) signed the informed consent form. The exclusion criteria were (1) cognitive deficits or (2) questionnaires with missing, wrong, or uncorrectable information. The study was approved by the Ethics Committee of the Bayi Orthopaedic Hospital. Written informed consent was obtained from all participants before completing the survey.

### Questionnaire

The questionnaire was designed based on the available guidelines [16–22]. A questionnaire with four dimensions was self-administered and modified based on the advice from two experts in orthopedics, deleting some similar or repetitive questions and refining some questions that were not clearly formulated. A pre-test (37 copies) was conducted before the formal launch, showing Cronbach’s α = 0.846, suggesting a high degree of internal consistency.

The final questionnaire included (1) the demographic characteristics of the participants (including age, gender, residence, education, work status, income, etc.), (2) the knowledge dimension (including 12 questions on gouty arthritis, with a score of 2 points for very well known, 1 point for heard of, and 0 points for unclear), (3) the attitude dimension (containing eight questions, all using a 5-point Likert scale ranging from very positive [5 points] to very negative [1 point]), and (4) the practice dimension (consisting of 14 questions, also using a five-point Likert scale ranging from always (5 points) to never (1 point)). Higher scores indicated adequate knowledge, more positive attitudes, and more proactive practices. A score above 70% of the total score is considered good.

An online questionnaire was constructed using the Wen Juan Xing (WJX) platform (https://www.wjx.cn). A quick response (QR) code was generated to collect data via WeChat. The participants included in this study were permanent residents of Chengdu, and those aged 30 years old or older were included in this study. The participants were enrolled through convenience sampling. The study was advertised by posters and pamphlets in waiting rooms and community centers and through WeChat. The participants logged in and filled out the questionnaire by scanning the QR code from WeChat.

In order to ensure the quality and completeness of the questionnaire results, each IP address could only be used once to submit a questionnaire, and all items were mandatory. All questionnaires were checked for completeness, internal consistency, and reasonableness by the research team members. The questionnaires with a response time of < 2 min were excluded. For questionnaires with errors in logic or information, the respondents were contacted to confirm and correct their responses.

### Sample size

The formula

$$n={\left(\frac{{Z}_{1-\alpha /2}}{\delta }\right)}^{2}\times p\times \left(1-p\right)$$

can be used to calculate the sample size of cross-sectional surveys. In the formula, *n* represents the sample size for each group, *α* represents the type I error (which is typically set at 0.05), *Z*_*1−α/2*_=1.96, *δ* represents the allowable error (typically set at 0.05), and *p* is set at 0.5 (as setting it at 0.5 maximizes the value and ensures a sufficiently large sample size). Hence, the calculated sample size was 384. Considering an estimated questionnaire response rate of 80%, 480 valid questionnaires were needed.

### Statistical analysis

The statistical analysis software was Stata 17.0 (Stata Corporation, College Station, TX, USA). The continuous indicators were described as means ± standard deviation (SD) and analyzed using Student’s t-test or one-way ANOVA. The categorical indicators were described using n (%). Logistic regression was used to conduct univariable and multivariable analyses of knowledge, attitudes, and practices, and 70% of the total scores were used as the cut-off values. Variables with *P* < 0.05 in the univariable variables were included in the multivariable analysis. A structural equation modeling (SEM) analysis was performed to examine the relationships among the KAP dimensions. It was hypothesized that knowledge directly affected attitudes and practices and indirectly affected practices, while attitudes directly influenced practices. Two-sided *P*-values < 0.05 were considered statistically significant.

## Results

### Demographic characteristics

A total of 671 questionnaires were collected. Based on the exclusion criteria, 102 people had missing or wrong information that could not be corrected, and 32 people disagreed with being disclosed, resulting in the inclusion of 537 questionnaires in the analysis. The participants were 40.04 ± 11.93 years of age. Most participants were male (52.51%), with a body mass index (BMI) of 18.5–23.9 kg/m^2^ (64.25%), living in urban areas (64.62%), with a junior college or undergraduate education (51.77%), heads of institution/organization and professional and technical staff (32.03%), with an income of 2,000–5,000 yuan (44.32%), married (77.47%), and with medical insurance (93.85%) (Table 1).

**Table 1:** Baseline characteristics and KAP scores

Variables	Knowledge score			Attitude score		Practice score	
	*N* (%)	Mean ± SD	*P*	Mean ± SD	*P*	Mean ± SD	*P*
Total	537	13.12 ± 6.41		25.28 ± 3.97		45.25 ± 5.77	
Gender			< 0.001		< 0.001		< 0.001
Male	282 (52.51)	14.23 ± 6.30		24.73 ± 3.95		44.29 ± 5.29	
Female	255 (47.49)	11.88 ± 6.32		25.89 ± 3.90		46.30 ± 6.11	
Age	40.04 (11.93)		-		-		-
Body mass index (kg/m^2^)			0.674		0.337		0.123
< 18.5	26 (4.84)	12.65 ± 5.61		24.92 ± 3.90		44.92 ± 4.24	
18.5–23.9	345 (64.25)	13.35 ± 6.40		25.14 ± 4.04		45.57 ± 5.99	
24–28	128 (23.84)	12.83 ± 6.41		25.84 ± 3.86		45.01 ± 5.49	
> 28	38 (7.08)	12.26 ± 7.16		24.97 ± 3.62		43.29 ± 5.37	
Residence			0.002		0.035		0.052
Rural	154 (28.68)	12.75 ± 6.71		24.59 ± 3.80		44.29 ± 5.69	
Urban	347 (64.62)	13.63 ± 6.29		25.54 ± 4.04		45.64 ± 5.66	
Suburban	36 (6.70)	9.72 ± 5.16		25.75 ± 3.64		45.56 ± 6.81	
Education			< 0.001		0.001		0.470
Middle school and below	132 (24.58)	11.23 ± 6.90		24.35 ± 3.49		44.80 ± 5.58	
High school/technical secondary school	127 (23.65)	13.72 ± 6.14		25.05 ± 3.78		45.09 ± 5.57	
College and above	278 (51.77)	13.74 ± 6.13		25.83 ± 4.18		45.53 ± 5.96	
Occupation			< 0.001		0.036		0.049
Heads of institution/organization and professional and technical staff	172 (32.03)	15.55 ± 5.74		25.47 ± 4.43		45.79 ± 6.29	
General employees	139 (25.88)	12.71 ± 6.39		24.73 ± 3.61		44.67 ± 5.57	
Commercial and service industry personnel	79 (14.71)	12.20 ± 5.92		24.81 ± 3.84		44.48 ± 5.16	
Production personnel and transport personnel	50 (9.31)	13.22 ± 6.40		25.02 ± 3.96		44.12 ± 4.51	
Other	97 (18.06)	10.07 ± 6.48		26.26 ± 3.54		46.31 ± 5.99	
Monthly per capita income, yuan			< 0.001		< 0.001		0.128
< 2000	64 (11.92)	10.14 ± 5.66		24.77 ± 3.07		44.47 ± 6.17	
2000–5000	238 (44.32)	13.41 ± 6.47		24.47 ± 3.73		44.84 ± 5.27	
5000–10,000	158 (29.42)	13.85 ± 6.02		26.31 ± 4.36		46.08 ± 5.89	
> 10,000	77 (14.34)	13.17 ± 7.00		26.13 ± 3.91		45.42 ± 6.53	
Marital status			0.498		< 0.001		0.615
Unmarried	91 (16.95)	12.45 ± 6.85		26.88 ± 4.09		45.49 ± 5.60	
Married	416 (77.47)	13.29 ± 6.27		25.00 ± 3.84		45.26 ± 5.76	
Others	30 (5.59)	12.73 ± 7.01		24.37 ± 4.25		44.30 ± 6.57	
Type of medical insurance			< 0.001		< 0.001		0.003
Social medical insurance only	308 (57.36)	12.73 ± 6.46		25.28 ± 3.86		45.05 ± 5.73	
Commercial medical insurance only	83 (15.46)	15.27 ± 5.17		23.46 ± 3.4		43.83 ± 4.49	
Both social and commercial medical insurance	113 (21.04)	13.99 ± 6.28		26.51 ± 4.18		46.81 ± 6.26	
No medical insurance	33 (6.15)	8.36 ± 6.49		25.67 ± 3.91		45.27 ± 6.30	

### Knowledge, attitudes, and practices

The mean knowledge, attitudes, and practices scores were 13.12 ± 6.41 (possible range: 0–24), 25.28 ± 3.97 (possible range: 8–40), and 45.25 ± 5.77 (possible range: 14–70), respectively, indicating inadequate knowledge, more unfavorable attitudes and less proactive practices (Table 1). Around 54.93% are aware that high blood uric acid can cause joint and tissue damage. Additionally, 49.35% know that a high uric acid level can be asymptomatic but may suddenly flare up due to factors like alcohol consumption or consumption of large amounts of purine-containing foods. Symptoms of gouty arthritis, such as redness, swelling, heat, and joint pain, are recognized by 55.31% of participants. However, only 39.66% know that gout pain can resolve on its own within a few days or two weeks. Awareness of the association between gout/hyperuricemia and chronic diseases stands at 29.24%. Lifestyle factors like high-calorie diets and alcohol consumption are known to contribute to gout by 51.21% of participants. The importance of dietary modifications, including consuming fruits, vegetables, nuts, legumes, low-fat dairy products, and whole grains/mixed grains to reduce gout incidence, is understood by 35.38% of participants. Furthermore, 49.91% recognize that excessive intake of high-purine foods like animal meats and seafood can increase gout attacks (Supplementary Table 1).

A majority of participants (50.23%) strongly agree that gouty arthritis is painful and causes anxiety. However, only a small percentage (15.28%) agree on the importance of careful dietary intake for prevention. Misconceptions exist, with 39.94% believing that avoiding simultaneous consumption of beer and seafood prevents gout. Furthermore, a majority (58.4%) believe that gouty arthritis is a lifelong incurable disease. On the positive side, 55.52% recognize the importance of lifestyle improvements and active management for controlling gout and hyperuricemia. Regarding treatment, a significant number of participants (19.67%) would consider forgoing treatment due to cost, relying solely on a diet. Additionally, 18.76% would discontinue treatment if symptoms persisted (Supplementary Table 2).

Several behaviors, such as frequent alcohol consumption (24.02%), carbonated drinks and fruit juice intake (26.26%), excessive seafood consumption (25.51%), and high meat intake (32.03%), show non-conformity. Conversely, participants conform to drinking plenty of water (32.96%) and consuming recommended amounts of eggs, vegetables, and low-fat milk (ranging from 25.14 to 42.09%). In non-dietary behaviors, conformity is observed in regular medical check-ups (38.18%), active physical activity and weight control (41.34%), and seeking prompt medical attention for joint symptoms (56.42%) (Supplementary Table 3).

### Multivariable logistic regression analysis

Multivariable logistic regression analysis showed that female (OR = 0.47, 95%CI: 0.31–0.71, *P* < 0.001), suburban living (OR = 0.18, 95%CI: 0.04–0.78, *P* = 0.022), heads of institution/organization and professional and technical staff (OR = 2.04, 95%CI: 1.23–3.39, *P* = 0.006), and an income of < 2,000 yuan (OR = 0.35, 95%CI: 0.14–0.85, *P* = 0.021) were independently associated with knowledge. Female (OR = 2.17, 95%CI: 1.43–3.30, *P* < 0.001), age (OR = 1.03, 95%CI: 1.01–1.05, *P* = 0.001), college and above education (OR = 2.26, 95%CI: 1.16–4.41, *P* = 0.017), an income of 5,000–10,000 yuan (OR = 2.05, 95%CI: 1.27–3.31, *P* = 0.003), and an income of > 10,000 yuan (OR = 2.07, 95%CI: 1.12–3.81, *P* = 0.020) were independently associated with attitudes. Attitude (OR = 1.31, 95%CI: 1.23–1.40, *P* < 0.001), female (OR = 1.62, 95%CI: 1.01–2.58, *P* = 0.044), and age (OR = 1.02, 95%CI: 1.00-1.04, *P* = 0.016) were independently associated with practices (Table 2).

**Table 2:** Multivariable logistic regression analysis

	Variables	Multivariable analysis	
		OR (95%CI)	*P*
Knowledge	Gender		
	Male	Ref.	
	Female	0.47 (0.31, 0.71)	< 0.001
	Residence		
	Rural	1.19 (0.73, 1.94)	0.479
	Urban	Ref.	
	Suburban	0.18 (0.04, 0.78)	0.022
	Occupation		
	Heads of institution/organization and professional and technical staff	2.04 (1.23, 3.39)	0.006
	General employees	Ref.	
	Commercial and service industry personnel	0.70 (0.35, 1.40)	0.311
	Production personnel and Transport personnel	1.39 (0.63, 3.04)	0.412
	Other	0.51 (0.23, 1.10)	0.087
	Monthly per capita income, yuan		
	< 2000	0.35 (0.14, 0.85)	0.021
	2000–5000 ref	Ref.	
	5000–10,000	0.79 (0.49, 1.28)	0.341
	> 10,000	0.58 (0.31, 1.09)	0.092
	Type of medical insurance		
	Social medical insurance only	Ref.	
	Commercial medical insurance only	1.36 (0.79, 2.34)	0.272
	Both social and commercial medical insurance	0.93 (0.55, 1.58)	0.788
	No medical insurance	0.55 (0.15, 1.99)	0.363
Attitude	Knowledge	1.00 (0.96, 1.03)	0.875
	Gender		
	Male	Ref.	
	Female	2.17 (1.43, 3.30)	< 0.001
	Age	1.03 (1.01, 1.05)	0.001
	Residence		
	Rural	0.87 (0.52, 1.47)	0.614
	Urban	Ref.	
	Suburban	0.95 (0.43, 2.13)	0.908
	Education		
	Middle school and below	Ref.	
	High school/technical secondary school	1.82 (0.95, 3.49)	0.071
	College and above	2.26 (1.16, 4.41)	0.017
	Occupation		
	Heads of institution/organization and professional and technical staff	1.36 (0.77, 2.41)	0.287
	General employees	Ref.	
	Commercial and Service industry personnel	1.38 (0.70, 2.74)	0.354
	Production personnel and transport personnel	2.23 (0.96, 5.18)	0.063
	Other	1.92 (0.99, 3.72)	0.053
	Monthly per capita income		
	< 2000	0.50 (0.22, 1.11)	0.088
	2000–5000	Ref.	
	5000–10,000	2.05 (1.27, 3.31)	0.003
	> 10,000	2.07 (1.12, 3.81)	0.020
	Type of medical insurance		
	Social medical insurance only	Ref.	
	Commercial medical insurance only	0.49 (0.24, 0.99)	0.047
	Both social and commercial medical insurance	1.59 (0.97, 2.62)	0.067
	No medical insurance	1.91 (0.0.81, 4.52)	0.141
Practice	Knowledge	0.99 (0.95, 1.02)	0.498
	Attitudes	1.31 (1.23, 1.40)	< 0.001
	Gender		
	Male	Ref.	
	Female	1.62 (1.01, 2.58)	0.044
	Age	1.02 (1.00, 1.04)	0.016
	Body mass index (kg/m^2^)		
	< 18.5	0.52 (0.15, 1.75)	0.293
	18.5–23.9	Ref.	
	24–28	0.64 (0.37, 1.11)	0.114
	> 28	0.37 (0.14, 1.01)	0.052
	Occupation		
	Heads of institution/organization and professional and technical staff	0.95 (0.52, 1.75)	0.879
	General employees	Ref.	
	Commercial and service industry personnel	0.60 (0.27, 1.30)	0.195
	Production personnel and transport personnel	0.42 (0.16, 1.11)	0.080
	Other	1.80 (0.91, 3.56)	0.091
	Monthly per capita income		
	< 2000	0.91 (0.42, 1.95)	0.802
	2000–5000	Ref.	
	5000–10,000	1.20 (0.69, 2.09)	0.515
	>10,000	1.33 (0.67, 2.61)	0.413
	Type of medical insurance		
	Social medical insurance only	Ref.	
	Commercial medical insurance only	0.91 (0.44, 1.90)	0.808
	Both social and commercial medical insurance	1.69 (0.96, 2.95)	0.067
	No medical insurance	0.88 (0.33, 2.32)	0.796

OR: odds ratio; CI: confidence interval

### Structural equation modeling

**Fig. 1:**
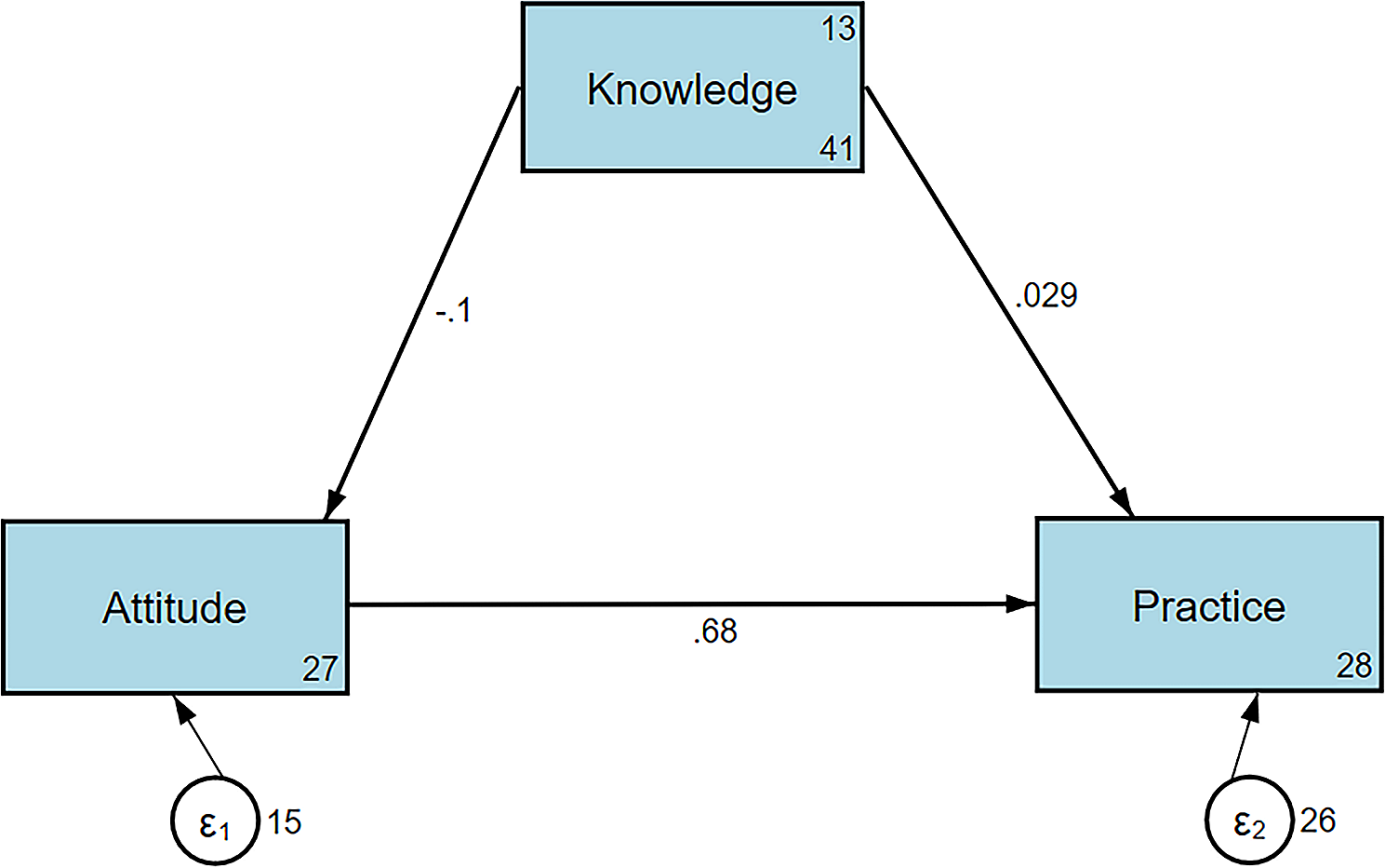
Structural equation modeling

The SEM showed that knowledge had a significant negative direct effect on attitude (β=-0.10, *P* < 0.001). Attitude, in turn, had a strong positive direct effect on practice (β = 0.68, *P* < 0.001). However, the direct effect of knowledge on practice was not significant (β = 0.03, *P* = 0.412). There was an indirect effect from knowledge to practice through attitude (β=-0.07, *P* < 0.001) (Fig. 1 and Supplementary Table 4).

## Discussion

The general population over 30 years old had inadequate knowledge, more unfavorable attitudes, and less proactive practices toward gouty arthritis. This study is the first from a Chinese population. Gout is a relatively common disease with high morbidity, but it can be prevented or delayed with proper lifestyle habits. In addition, early consultation, diagnosis, and management can improve prognosis. The present study suggests targeted interventions are necessary to enhance knowledge and promote positive attitudes toward gouty arthritis among individuals aged over 30. These interventions should focus on education, addressing misconceptions, reducing stigmas, and emphasizing early diagnosis and adherence to management strategies. Tailoring interventions to high-risk groups, collaborating with healthcare professionals, increasing accessibility to information, and monitoring progress are key to improving practices.

The development of gouty arthritis is a complex trait influenced by non-modifiable risk factors (e.g., genetics and uric acid production and elimination) and by modifiable factors (e.g., high-fructose and high-purine diet, medications, and obesity) [2, 3]. Therefore, gouty arthritis is partly preventable, but its prevention relies on good life habits. Still, those life habits must be known to be applied properly. Females had a lower knowledge of gouty arthritis, possibly because gout and gouty arthritis are mostly prevalent in males, who also display a higher prevalence of gout-associated life habits, like alcohol drinking and smoking [23, 24]. On the other hand, the female gender was associated with higher attitude and practice scores, possibly because of the lower prevalence of alcohol drinking and smoking, which are life habits that are difficult to break [23, 24]. In this study, a higher socioeconomic status was generally associated with a higher KAP. Socioeconomic status is well-known to influence health literacy [25]. Individuals with a higher socioeconomic status often have higher education, work in professional domains that require high knowledge (like healthcare, finances, and law), and have access to resources that require payment (e.g., private libraries, magazines, etc.) for example. All of the above can contribute to a higher exposure of the individuals to bits of knowledge about gout.

The present study showed poor KAP toward gouty arthritis in Chengdu (China). Those results are supported by a study in Pakistan that revealed that several patients were suffering from gout without knowing it was gout and that there were available treatments [15]. Physicians also display gaps in knowledge about gout [12–14]. Physicians are a primary source of health-related information [26], but if they have gaps in knowledge, they will be unable to pass on the appropriate information. The present study did not specifically include healthcare professionals, but examining their KAP toward gout in future studies would be interesting.

In this study, knowledge directly influenced attitude but not practice, while attitude directly influenced practice. This suggests that improving knowledge about gouty arthritis can lead to more positive attitudes toward the condition. However, translating these attitudes into proactive practices might require additional interventions or strategies. To address this, targeted interventions should be implemented, focusing on education, dispelling misconceptions, reducing stigmas, and emphasizing early diagnosis and adherence to management strategies. It is important to tailor these interventions to high-risk groups, collaborate with healthcare professionals, improve accessibility to information, and monitor progress to foster improved practices.

This study has limitations. It was performed in a single area, resulting in a relatively small sample size. The cross-sectional design prevented the determination of causality. Nevertheless, this study could be used as a baseline to evaluate the effect of future interventions. The participants had a relatively high socioeconomic status, which was not completely representative of the Chinese population. Finally, all KAP surveys are at risk of social desirability bias, in which the participants are tempted to answer what they should do instead of what they are doing [27, 28]. Still, considering that the attitude and practice scores were relatively low, the likelihood of that bias is low here.

In conclusion, the general population aged over 30 exhibited insufficient knowledge, unfavorable attitudes, and a lack of proactive practices concerning gouty arthritis. To address this issue, targeted interventions should prioritize improving knowledge about gout and fostering positive attitudes toward its management.

### Electronic supplementary material

Below is the link to the electronic supplementary material.

Supplementary Material 1

Supplementary Material 2

## Data Availability

All data generated or analyzed during this study are included in this published article [and its supplementary information files].
