# Supplementary material for: Knowledge, attitudes, and practices of gouty arthritis in the general population aged > 30
Source: BMC Med Educ. 2024 Jul 19;24:775. doi: 10.1186/s12909-024-05690-x (PMC11264954; doi:10.1186/s12909-024-05690-x)
Supplement: Supplementary file 1 — Supplementary Material 1 [file 12909_2024_5690_MOESM1_ESM.docx]

**Supplementary Table 1.** Knowledge

| Knowledge | Very well known | Heard of | Unclear |
| --- | --- | --- | --- |
| 1. Uric acid is the end product of purine metabolism in the body, but a high blood uric acid level can cause damage by depositing in the joints and tissues. | 171 (31.84) | 295 (54.93) | 71 (13.22) |
| 2. A state of high blood uric acid level can be asymptomatic but can suddenly flare up after a full meal and alcohol consumption, excessive fatigue, stress, localized injury to a joint, surgery, exposure to cold and moisture, etc. | 154 (28.68) | 265 (49.35) | 118 (21.97) |
| 3. Gouty arthritis often begins in the first metatarsophalangeal joint of the foot, and the foot arch, ankle joints, knee joints, wrist joints, and elbow joints are also common sites of attack. | 170 (31.66) | 258 (48.04) | 109 (20.3) |
| 4. Gouty arthritis is one of the main manifestations of patients with gout and is mainly characterized by redness, swelling, heat, and joint pain. | 162 (30.17) | 297 (55.31) | 78 (14.53) |
| 5. Many of the pain caused by acute gouty arthritis may resolve on its own within a few days or two weeks. | 160 (29.8) | 213 (39.66) | 164 (30.54) |
| 6. Most patients suffer from more joint injuries and frequent gout attacks due to the failure to keep hyperuricemia under control for a prolonged period. | 148 (27.56) | 259 (48.23) | 130 (24.21) |
| 7. As the disease progresses uncontrolled, it may eventually lead to joint deformities, the presence of gout nodules or gouty tophi, and even gouty nephropathy. | 165 (30.73) | 257 (47.86) | 115 (21.42) |
| 8. Gouty tophi can be as small as a sesame or as large as an egg or larger, which can break down or form a fistula when it is squeezed, with a white bean-like discharge | 136 (25.33) | 243 (45.25) | 158 (29.42) |
| 9. Gout/hyperuricemia is closely associated with the onset and development of many chronic diseases, such as metabolic diseases, cardiovascular diseases, and renal diseases. | 157 (29.24) | 236 (43.95) | 144 (26.82) |
| 10. Gout is a lifestyle-related disease closely associated with a long-term high-calorie diet and heavy alcohol consumption. | 163 (30.35) | 275 (51.21) | 99 (18.44) |
| 11. A high intake of fruits, vegetables, nuts, legumes, low-fat dairy products, and whole grains/mixed grains can significantly reduce the incidence of gout. | 190 (35.38) | 252 (46.93) | 95 (17.69) |
| 12. Excessive intake of high-purine foods such as animal meats, seafood, hot pot, and thick soups can increase the incidence of gout attacks. | 187 (34.82) | 268 (49.91) | 82 (15.27) |

**Supplementary Table 2.** Attitudes

| Attitude | Strongly agree | Agree | Neutral | Disagree | Strongly disagree |
| --- | --- | --- | --- | --- | --- |
| 1. Gouty arthritis is painful and unbearable, and I feel anxious about the possibility of developing it. N | 149 (50.23) | 245 (29.95) | 114 (16.04) | 24 (0.76) | 5 (3.03) |
| 2. It is important to be careful with your daily dietary intake to prevent gouty arthritis. P | 183 (15.28) | 262 (7.87) | 72 (13.01) | 14 (41.45) | 6 (22.39) |
| 3. You will not get gout if you don’t drink beer and eat seafood simultaneously. N | 74 (39.94) | 116 (39.64) | 109 (14.98) | 179 (2.57) | 59 (2.87) |
| 4. Gouty arthritis is a lifelong disease that can never be cured. N | 88 (58.4) | 181 (28.29) | 125 (9.83) | 127 (1.36) | 16 (2.12) |
| 5. Similar to managing other chronic metabolic diseases, lifestyle improvement and active integrated management for controlling risk factors are very important to control gout and hyperuricemia. P | 149 (55.52) | 268 (31.47) | 87 (10.29) | 27 (0.91) | 6 (1.82) |
| 6. In an acute attack, it is important to seek medical attention and give medication to control the inflammation as soon as possible; even if you know it will resolve on its own within a week, you should not just put up with it. P | 135 (24.81) | 263 (30.11) | 84 (29.95) | 45 (9.98) | 10 (5.14) |
| 7. If I was diagnosed with gouty arthritis/hyperuricemia, I would probably give up on being treated if it was too expensive and just be careful with my daily diet. N | 90 (19.67) | 173 (31.16) | 109 (31.32) | 137 (13.16) | 28 (4.69) |
| 8. If I were diagnosed with gouty arthritis/hyperuricemia, I would probably give up the treatment if my symptoms did not resolve after a period of treatment. N | 73 (18.76) | 166 (24.81) | 94 (31.47) | 170 (19.06) | 34 (5.9) |

**Supplementary Table 3.** Practice

| Practice | a. Very conforming | b. Conforming | c. Neutral | d. Not conforming | e. Very not conforming |
| --- | --- | --- | --- | --- | --- |
| 1. Frequent alcohol consumption (especially yellow wine, beer, and white wine). N | 78 (14.53) | 129 (24.02) | 114 (21.23) | 118 (21.97) | 98 (18.25) |
| 2. Carbonated drinks, fruit juice drinks. N | 80 (14.9) | 141 (26.26) | 148 (27.56) | 120 (22.35) | 48 (8.94) |
| 3. Seafood (more than 500 g per week). N | 66 (12.29) | 137 (25.51) | 138 (25.7) | 132 (24.58) | 64 (11.92) |
| 4. Meat (especially beef, lamb, and pork, more than 200 g per day). N | 106 (19.74) | 172 (32.03) | 175 (32.59) | 71 (13.22) | 13 (2.42) |
| 5. Thick meat soups, fish soups. N | 81 (15.08) | 136 (25.33) | 177 (32.96) | 118 (21.97) | 25 (4.66) |
| 6. Animal offal. N | 67 (12.48) | 129 (24.02) | 168 (31.28) | 138 (25.7) | 35 (6.52) |
| 7. Plenty of drinking water (more than 2000 ml). P | 85 (15.83) | 177 (32.96) | 185 (34.45) | 67 (12.48) | 23 (4.28) |
| 8. Eggs (one per day). P | 137 (25.51) | 173 (32.22) | 135 (25.14) | 78 (14.53) | 14 (2.61) |
| 9. Vegetables (500 g per day). P | 126 (23.46) | 226 (42.09) | 144 (26.82) | 37 (6.89) | 4 (0.74) |
| 10. low-fat, skimmed milk (300 ml per day). P | 97 (18.06) | 170 (31.66) | 176 (32.77) | 77 (14.34) | 17 (3.17) |
| 11. Late-night eating/snacking. N | 67 (12.48) | 115 (21.42) | 162 (30.17) | 144 (26.82) | 49 (9.12) |
| To what extent do you conform to the following behaviors: | | | | | |
| 12. Regular medical check-ups. P | 108 (20.11) | 205 (38.18) | 134 (24.95) | 74 (13.78) | 16 (2.98) |
| 13. Active physical activity and weight control. P | 84 (15.64) | 164 (30.54) | 222 (41.34) | 55 (10.24) | 12 (2.23) |
| 14 Seek prompt medical attention if there is redness, swelling, and pain in joints. P | 102 (18.99) | 303 (56.42) | 88 (16.39) | 39 (7.26) | 5 (0.93) |

**Supplementary Table 4.** Parameters of the SEM analysis

| Model paths | Direct effect |  | Indirect effect |  |
| --- | --- | --- | --- | --- |
|  | β (95% CI) | P | β (95% CI) | P |
| K→A | -0.10 (-0.15, -0.05) | <0.001 |  |  |
| A→P | 0.68 (0.57, 0.79) | <0.001 |  |  |
| K→P | 0.03 (-0.04, 0.10) | 0.412 | -0.07 (-0.11, -0.03) | <0.001 |

CI: confidence interval; K: knowledge; A: attitudes; P: practice.
